# Effects of *Ficus carica* L. polysaccharide on the intestinal immune function and microbiota of broilers

**DOI:** 10.3389/fimmu.2025.1579046

**Published:** 2025-04-08

**Authors:** Yabin Lu, Sajidaimu Maimaiti, Zhanke Qin, Xinke Cheng, Jianlong Li, Chuang Zhou, Ying Xiao, Saifuding Abula, Ling Kuang, Zhanhai Mai

**Affiliations:** ^1^ College of Veterinary Medicine, Xinjiang Agricultural University, Urumqi, Xinjiang, China; ^2^ Department of Agricultural Economics, Kezilesu Vocational and Technical College, Atushi, Xinjiang, China

**Keywords:** *Ficus carica* L. polysaccharides, chicken growth performance, network pharmacology, intestinal immunity, intestinal microbiota

## Abstract

**Introduction:**

Ficus carica L. polysaccharides (FLPs) are groups of biologically active compounds extracted from Ficus carica L.

**Methods:**

In this study, we analyzed the structure of FLPs, predicted their immune enhancement pathway, and detected the impact of FLPs on the growth performance, immune function, and intestinal microflora of broiler chickens.

**Results:**

The results showed that FLPs are comprised of monosaccharides including rhamnose, arabinose, mannose, glucose, and galactose. Feeding with FLPs significantly promoted the growth performance, slaughtering performance, and immune organs index of chickens compared to the control group (p < 0.05). Moreover, the FLP-h and FLP-m groups had increased levels of sIgA, IgG, IL-4, IL-5, IL-12, and IFN-g; improved immunity and barrier function; and a higher percentage of spleen CD4+ and CD8+ T cell differentiation compared to the control group (p < 0.05). Additionally, the FLP-h group had increased levels of various SCFAs, and increased beneficial bacteria such as Firmicutes at the phylum level and Faecalibacterium, Blautia, Phascolarctobacterium, and Alistipes at the genus level. The results of network pharmacology and KEGG pathway prediction indicate that FLPs may change the structure and metabolism of intestinal microbiota by enhancing carbon fixation pathways in prokaryotes, and promote intestinal immune barrier function through the joint action of bisphenol degradation, retinol metabolism, NODlike signaling pathways, toll-like receptor signaling pathways, and the MAPK signaling pathway.

**Discussion:**

These results suggest that FLP-h supplementation effectively promotes growth performance and enhances the intestinal mucosal immune barrier function in chickens.

## 1 Introduction

Intestinal health plays a pivotal role in ensuring optimal growth and developmental processes in both animals and birds. The intestinal well-being of poultry is evaluated through multiple parameters, encompassing gut microbiota composition, intestinal immune status, nutritional absorption, and environmental factors (1). In chicken farming, intestinal health problems are frequently caused by dysbiosis in the gut microbiota or inflammatory responses (2, 3). Chemicals such as antibiotics have been used effectively in poultry production over the past few years. However, using them extensively has a number of negative consequences. For example, the overuse of antibiotics has led to the development of antibiotic resistance in bacteria, which makes it more difficult to treat diseases (4). Some of these super-bugs have even become resistant to high-potency multiple antibiotics and pose a challenge for the healthcare system in effectively combating infectious diseases (5). The extensive application of antibiotics in livestock and poultry production has resulted in environmental accumulation, disturbance of microbial ecosystems, and contamination from pharmaceutical residues (6).

In traditional Chinese medicine, polysaccharides sourced from natural plants have received a lot of attention recently as an important bioactive element (7). Plant-derived polysaccharides function as natural immunity modulators. stimulate immune cells. and their interaction with the complement system releases cytokines (8, 9). These bioactive metabolites were essential for maintaining gut barrier function and general health (10, 11). Plant-derived polysaccharides may also improve intestinal microbial diversity. Administrating plant polysaccharides to animals effectively promotes the beneficial bacteria in the digestive system, which contribute to digestion, nutrient absorption, intestinal barrier function, and overall gut health (12). Studies have demonstrated that Astragalus polysaccharide (APS) supplementation significantly improved average daily gain, reduced the feed conversion ratio, promoted the expression of multiple cytokines, improved intestinal structure and intestinal flora balance of broilers, and showed good growth promotion and immune enhancement effects (8, 13). Because of its good immune-enhancing activity, APS is usually used as an immune-enhancing agent in livestock and poultry production. Furthermore, supplementation with prebiotic Dendrobium huoshanense polysaccharides can enhance the health of the host by affecting the ecological balance of gut bacteria and modifying gastrointestinal immune function (14). These results imply that polysaccharide-containing nutritional supplementation may increase gut immunity and microbial balance, both of which contribute to improved host health.

*Ficus carica* L. is a fig genus plant in the mulberry family. *Ficus carica* L. (Moraceae, fig tree) is widely distributed in the warmer Mediterranean and sub-Mediterranean regions in the Northern Hemisphere and also in many regions of the Southern Hemisphere (15). In China, Xinjiang province is a core production area of *Ficus carica* L. Previous studies have found that *Ficus carica* L. are abundant in polysaccharides, flavonoids, polyphenols, and coumarins, of which *Ficus carica* L. polysaccharides showed good antioxidant and detumescence effects to improve digestion and enhance immunity (16). Du et al. extracted a novel polysaccharide FCPW80-2 from *Ficus carica* L. with a backbone composed of α-L-Ara linked in a 1→5 configuration, β-D-Man linked in a 1→3,6 configuration, and β-D-Gal linked in a 1→4,6 configuration. FCPW80-2 can promote phagocytosis of RAW264, secretion of NO, TNF-α, and IL-6, and showed good cellular immune enhancement activity (17). Another study demonstrated that *Ficus carica* L. polysaccharides are readily broken down and consumed by the gut microbiota and promote intestinal health and prevent the underlying disease, which also greatly lowers the number of dangerous bacteria and raises the numbers of short-chain fatty acids (SCFAs) (17).

*Ficus carica* L. is a common food for domestic chickens, but the impact of *Ficus carica* L. on the growth performance and immune function of chickens has not been studied yet. Therefore, the main polysaccharides (FLPs) from *Ficus carica* L. were isolated, and spectroscopic and chromatographic techniques were employed to characterize their functional compounds and monosaccharide composition. The effects of FLPs on growth performance, antibody production, cytokine profiles, spleen T cell differentiation (CD4^+^/CD8^+^ ratio), immune organ histology, intestinal barrier integrity, gut microbiota composition, SCFA concentrations, and associated signaling pathways were systematically evaluated.

## 2 Materials and methods

### 2.1 Structural analysis of FLPs

The *Ficus carica* L. samples were collected in August. The samples were identified by fingerprint (S1). The crude polysaccharides from *Ficus carica* L. were obtained by employing water decoction and ethanol precipitation techniques, subsequently eliminating proteins using the Sevag method (18). A diethylaminoethyl cellulose-650M (DEAE-650M) column was used to further purify the raw polysaccharides. A distilled water solution and 0.2 and 0.4 M NaCl were used for elution, correspondingly. The sugar content and aldoxic acid content in FLPs were detected using the phenol sulfuric acid method and sulfate-carbazole method, respectively. The physical structures of FLPs were examined via scanning electron microscopy (SEM), the functional groups were characterized by Fourier-transform infrared spectroscopy (FT-IR), and the monosaccharide composition was analyzed by gas chromatography (GC) following acid hydrolysis and derivatization with trifluoroacetic acid (TFA), as detailed in a previous study (19).

### 2.2 Target genes screening of FLP

The monosaccharides rhamnose, arabinose, mannose, glucose, and galactose in FLP were the keywords that were searched for in the databases of the Bioinformatics Analysis Tool for Molecular Mechanism of Traditional Chinese Medicine (BATMAN TCM) analytical platform and the Traditional Chinese Medicine Systems Pharmacology Database. To find targets that strengthened the immune system, we combined the categories of targets from the two databases and eliminated the duplicates. The findings were sorted according to the criteria of bioavailability during oral administration (≥30%) and drug resemblance (≥0.18) to determine the key active components’ matching targets. Gene names were obtained using Perl programs. To find the genes linked to these disorders, we searched the online Mendelian Lineage in the Man and Gene Cards database using the phrase “immune enhancement.” To find the drug targets’ common targets, we created a Venn diagram by comparing the drug’s targets with immune-enhancing targets using the R programming language. To obtain appropriate information on biological procedures (BPs), components of cells (CCs), molecular functioning (MF), and the associated pathway from the KEGG databases prioritized on the basis of P-values, we entered the key/target protein into the DAVID database.

### 2.3 Experimental design for animals

The Animal Welfare Ethics Committee of Xinjiang Agricultural University gave authorization to all experimental protocols (Approval # 2022016). In total, 75 1-day-hatched Sanhuang broilers (purchased from Kaige Livestock Co., Ltd.) were acclimated for 7 days and then randomly assigned into five groups (each group with five cages and three chickens per cage). All experiment groups were given a regular baseline diet (Tiankang Biotech Co., Ltd.). The control (gavage 5.0 mL normal saline daily), FLP-high group (5.0 mL gavage of FLP 2.0 g/kg daily), FLP-mid group (5.0 mL gavage of FLP 1.0 g/kg daily), and FLP-low group (5.0 mL gavage of FLP 0.5 g/kg daily). The positive drug group (5.0 mL gavage of APS 1.0 g/kg daily; APS produced by Sichuan Dingjian Animal Pharmaceutical Co., Ltd.). Over the course of the 50-day trial, five hens were chosen at random from each group on day 50. The blood was collected from the hearts and then the animals were euthanized and the following investigations were carried out.

### 2.4 Growth performance and immune organ index

The daily feed intake and body weight at the experiment’s initiation and termination were recorded to calculate the following growth parameters: average body weight (ABW), average daily feed intake (ADFI), average daily weight gain (ADWG), and feed conversion ratio (F/G) (20). At the experimental terminus, immune organs (spleen, bursa of Fabricius, and thymus) were dissected and weighed to calculate organ indices using the standard formula: immune organ index (g/kg) = organ weight/body weight (21).

### 2.5 Determination of slaughtering performance

At 50 days, five chickens were randomly selected from each group to be euthanized after blood collection. The following parameters were calculated according to a previous study: slaughtering rate, eviscerated rate, chest muscle rate, leg muscle rate, abdominal fat percentage, drip loss rate, and cooking loss rate (20).

### 2.6 Histology of HE-stained slices of the immune organs

For fixation, the cecal tonsils, ileum, spleen, thymus, jejunum, and duodenum were submerged in 4% paraformaldehyde at 50 days. The tissues were then processed histologically to produce slices that were stained with hematoxylin and eosin (HE) so that changes in their histopathological morphology could be seen. The morphology of the duodenum, jejunum, and ileum was observed, and the number of intestinal intraepithelial lymphocytes (IELs) on five intestinal villi in each section were counted randomly. In addition, 10 villus heights (V) and crypt depths (C) were randomly measured on slices of duodenum, jejunum, and ileum.

### 2.7 Histology of the alcian blue-periodic acid-schiff stained slices of the ileum

The ileum samples were collected and fixed in 4% paraformaldehyde to prepare paraffin slices. The paraffin slices were stained with alcian blue (AB) and periodic acid-schiff (PAS) to prepare the AB-PAS slices. The AB-PAS slices were visualized with an inverted light microscope, and goblet cells on five intestinal villi in each section were counted randomly.

### 2.8 Impacts of FLPs on intestine IgA secretion

The small intestine sections were incubated with mouse anti-chicken secretory immunoglobulin A (sIgA) primary antibodies. After washing with PBS, fluorescent secondary antibodies were marked with Cy3-IgA for incubation, and DAPI dye solution was later added for incubation in the dark. After washing with PBS, it was incubated with autofluorescence quencher solution and then rinsed with running water for 10 minutes. The tablets were sealed with anti-fluorescence quenched tablets, photographed with a fluorescence microscope (ECLIPSE 80i Nikon), and analyzed using ImageJ software.

### 2.9 Determination of immunoglobulins and cytokines

One mL of whole blood was drawn at 50 days, left to stand at 4°C for 1 hour, and the serum was separated by centrifuge at 2,000 rpm for 15 min. A 2 cm segment of the jejunum was excised and rinsed thoroughly with 1 mL of PBS buffer. The wash fluid was collected and centrifuged at 3,000 rpm for 15 minutes to obtain the supernatant. The total protein concentration in the supernatant was quantified using a BCA assay, and the concentration was normalized. Subsequently, the total sIgA content in the intestinal wash fluid was determined. ELISA kits were used to assess the serum IgG, serum cytokines (IL-4, IL-5, IL-12, and IFN-γ), and jejunum sIgA (Shanghai Kexing Trading Co, Ltd).

### 2.10 Flow cytometry analysis

At 50 d, the spleen was removed and then cut into pieces using a homogenizer to gather cells (Fluko Technology, Shanghai, China). Subsequently, the cells were stained with anti-chicken-CD3e, CD4, and CD8a, centrifuged, washed to remove the free antibodies, and analyzed using flow cytometry (BD Accuri C6^®^, USA).

### 2.11 Intestinal microbial 16s sequencing and SCFAs content detection

At 50 d, the cecal samples of feces were divided into two portions and stored in liquid nitrogen at a low temperature. Then, one portion was subjected to DNA quantification using Nanodrop. After conducting PCR amplification, the sample underwent purification to enable fluorescence quantification. The Nomi Biological Company created a sequencing library for computer-based sequencing analysis of intestinal bacteria. The other sample portions were subjected to boric acid extraction and the content of SCFAs was analyzed by gas chromatograph.

### 2.12 Statistical analysis

The statistical software SPSS 24.0 was utilized to conduct ANOVA for the analysis and data were provided as “X ± SE”. The Nomi Biological Company then created a sequencing library for a computer-based sequencing study of gut microbes (*p* < 0.05).

## 3 Results

### 3.1 Composition and structure of FLPs

**Figure 1:**
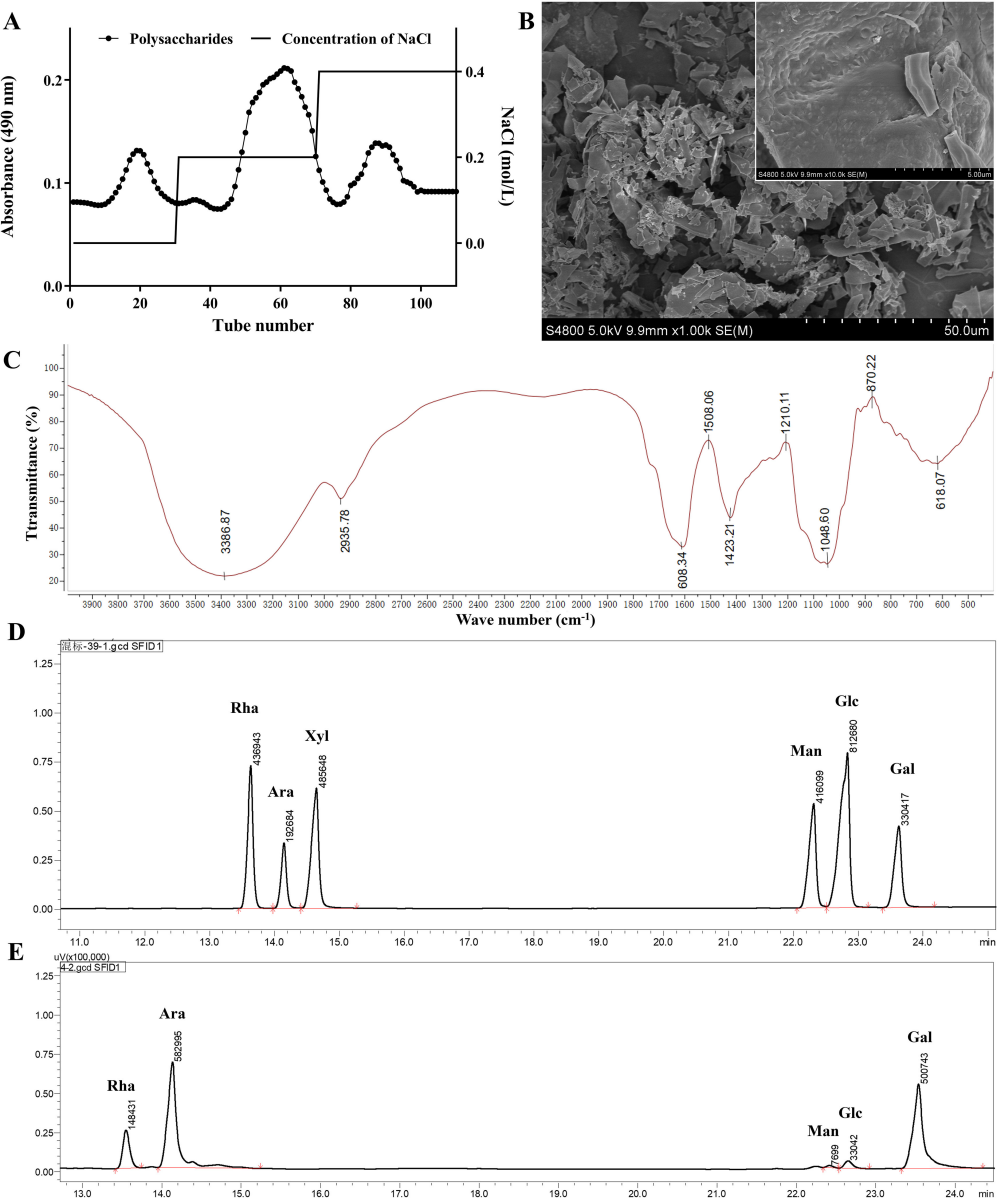
Characterization of FLPs. **(A)** Elution curve of FLPs, **(B)** scanning electron microscope images of FLPs, **(C)** FT-IR analysis of FLPs, **(D)** chromatogram of the monosaccharide standard, **(E)** monosaccharide composition of FLPs.

After ethanol precipitation and water extraction, purification was used to obtain the complete FLP using a DEAE column and elution with 0.2 mol/L NaCl to isolate the main polysaccharide fractions, which constituted 64.5% of the total polysaccharide content (**Figure 1A**). After separation and purification, the sugar content and aldehyde content in the FLPs were 74.75% and 17.35%, respectively. The SEM morphology of the FLPs, as shown in **Figure 1B**, revealed a smooth and dense textured structure. The FT-IR analysis results (depicted in **Figure 1C**) indicate that the intense absorption peak at 3,387cm^-^¹ is attributed to O-H stretching vibration, while the absorption peak at 2,936cm^-^¹ is due to C-H stretching vibration (10). Meanwhile, the peak at 1,608 cm^-1^ arises from the bending vibration of the hydroxyl group in the unbound carboxyl group of uronic acid. The C-H deformation vibration was subsequently expressed by the peak at 1,423 cm^-1^ (22). The peaks at approximately 1,200 and 1,000 cm^-1^ are from the pyranose ring (23). The GC analysis (**Figures 1D, E**) showed that the components of FLPs were rhamnose (11.66%), arabinose (45.80%), mannose (0.61%), glucose (2.60%), and galactose (39.34%). Arabinose and rhamnose were the predominant monosaccharides, suggesting that FLPs are acidic polysaccharides consisting of two sugars as the main backbone.

### 3.2 Target network construction for FLPs and immune enhancement

**Figure 2:**
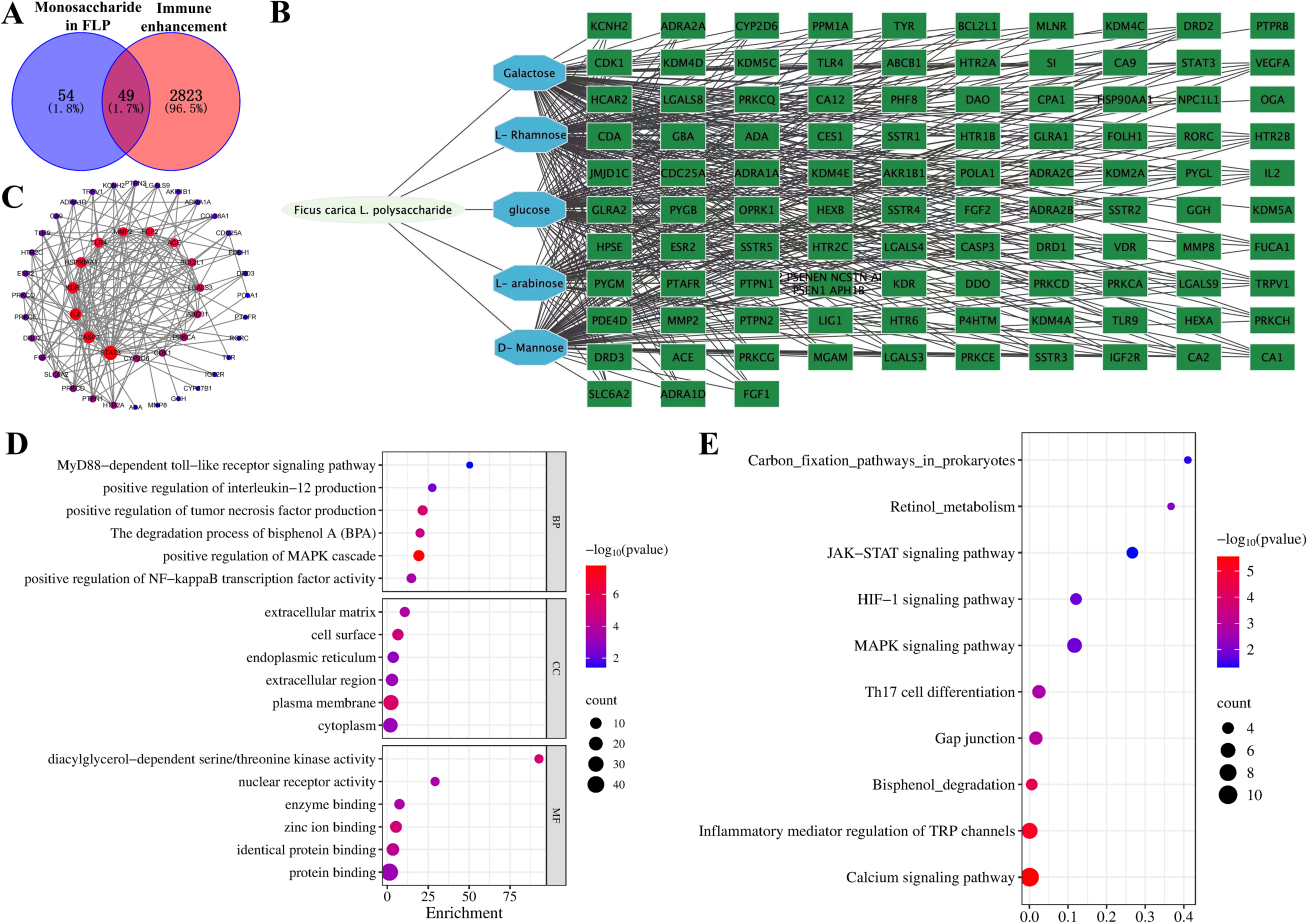
Exploring the role of FLP monosaccharide composition in immune enhancement based on the network pharmacology. **(A)** Venn diagram of FLP monosaccharide components and immune enhancement targets, **(B)** FLP monosaccharides exhibit the chain of interactions between the “component-target-disease” of immune enhancement, **(C)** PPI network of cross-targets, **(D)** GO function enrichment analysis of FLP monosaccharide components and immune enhancement, **(E)** enriched study of common targets using the KEGG pathway.

Network pharmacology methods were utilized to analyze the intersection of targets between the FLP monosaccharide and immune enhancement. The results showed that FLP monosaccharide has 103 target proteins, immune enhancement has 2872 target proteins, and the two groups had a total of 49 shared targets (**Figure 2A**). Using a string database, important targets were found for drug-disease targets through the establishment of a protein-protein interaction (PPI) network analysis (**Figure 2C**). There are 183 edges and 47 nodes in **Figure 2C**. The investigation revealed the protein interactions between the common targets of the monosaccharide in FLP and immune enhancement, including TLR4, FGF2, STAT3, and cytokine expression (**Figures 2B, C**). The intersection targets were found to be engaged in processes by GO enrichment analysis, such as MyD88-dependent toll-like receptor signaling pathway, positive regulation of IFN-β production, positive regulation of MAPK cascade, and the degradation process of bisphenol A (**Figure 2D**). Novel signaling pathways were discovered via KEGG enrichment analysis, comprising carbon fixation pathways in prokaryotes, the retinol metabolism, the JAK-STAT signaling pathway, the MAPK signaling pathway, Th17 cell differentiation, and the calcium signaling pathway (**Figure 2E**). The results indicate that FLPs intervene in immune enhancement by controlling the MAPK and the toll-like receptor signaling pathways, retinol metabolism, and the degradation process of bisphenol A to promote body immune function.

### 3.3 Effects of FLPs on the growth performance of broilers

The growth performance of broilers can be fully reflected by the ABW, ADWG, ADFI, and F/G indexes. As presented in **Table 1**, compared with the control group, ABW, ADWG, and ADFI in the FLP-h, FLP-m, and APS groups were significantly increased (*p* < 0.05), while there were no significant differences in ABW and ADWG between the FLP-h group and APS group (*p >*0.05). The F/G level in the FLP-h group showed a significant decrease compared to the FLP-m group, FLP-l group, APS group, and control group (*p* < 0.05).

**Table 1:** Impact of FLPs on the growth performance of broilers.

Group	ABW (g)	ADWG (g/d)	ADFI (g/d)	F/G (g/g)
FLP-h	1,850.73 ± 86.27^a^	41.13 ± 1.92^a^	116.20 ± 2.39^b^	2.83 ± 0.13^c^
FLP-m	1,566.53 ± 96.34^b^	34.81 ± 2.14^b^	114.20 ± 5.54^b^	3.23 ± 0.10^ab^
FLP-l	1,591.20 ± 79.19^b^	35.36 ± 1.76^b^	110.80 ± 4.82b^c^	3.19 ± 0.22^ab^
APS	1,865.73 ± 155.71^a^	35.36 ± 3.46^a^	126.80 ± 8.64^a^	3.06 ± 0.13^b^
Control	1,411.47 ± 32.30^c^	31.37 ± 0.72^c^	103.80 ± 4.60^c^	3.31 ± 0.18^a^

Different lowercase letters indicate significant differences (*p* < 0.05).

ABW, average body weight; ADWG, Average Daily Weight Gain; ADFI, Average Daily Feed Intake; F/G, Feed to Gain Ratio.

### 3.4 Effect of FLP on the slaughtering performance of broilers

As shown in **Table 2**, the slaughtering rate, eviscerated rate, chest muscle rate, and leg muscle rate of the FLP-h group and APS group were significantly higher than the control group (*p <*0.05). The values of the slaughtering rate and eviscerated rate in the FLP-m group were between the APS group and the control group. Compared to the control group, there was a significant increase in the percentage of abdominal fat, drip loss, and cooking loss in the FLP-h, FLP-m, and APS groups (*p <*0.05).

**Table 2:** Effect of FLPs on slaughter performance indexes of broiler chickens.

Group	FLP-h	FLP-m	FLP-l	APS	Control
Slaughtering rate (%)	97.46 ± 0.38^a^	96.76 ± 1.16^a^	96.36 ± 0.41^a^	97.12 ± 0.81^a^	93.94 ± 0.60^b^
Eviscerated rate (%)	76.67 ± 2.16^a^	75.12 ± 1.13^ab^	75.07 ± 2.70^ab^	77.73 ± 1.35^a^	73.71 ± 0.73^b^
Chest muscle rate (%)	17.19 ± 1.24^a^	15.36 ± 1.32^b^	14.84 ± 0.93^b^	17.35 ± 0.87^a^	11.15 ± 0.49^c^
Leg muscle rate (%)	17.62 ± 1.23^a^	15.23 ± 1.19^b^	14.84 ± 1.12^b^	17.60 ± 0.76^a^	11.74 ± 0.39^c^
Abdominal fat rate (%)	1.46 ± 0.07^c^	1.70 ± 0.17^b^	1.91 ± 0.18^b^	1.43 ± 0.15^c^	2.27 ± 0.15^a^
Drip loss rate (%)	1.35 ± 0.15^c^	1.52 ± 0.15^bc^	1.68 ± 0.08^b^	1.34 ± 0.09^c^	2.58 ± 0.30^a^
Cooking loss rate (%)	36.30 ± 1.10^c^	37.41 ± 1.49^c^	40.84 ± 1.21^b^	38.19 ± 0.63^c^	45.01 ± 1.60^a^

Different lowercase letters indicate significant differences (*p* < 0.05).

### 3.5 Analysis of HE-stained sections and immune organ index

**Figure 3:**
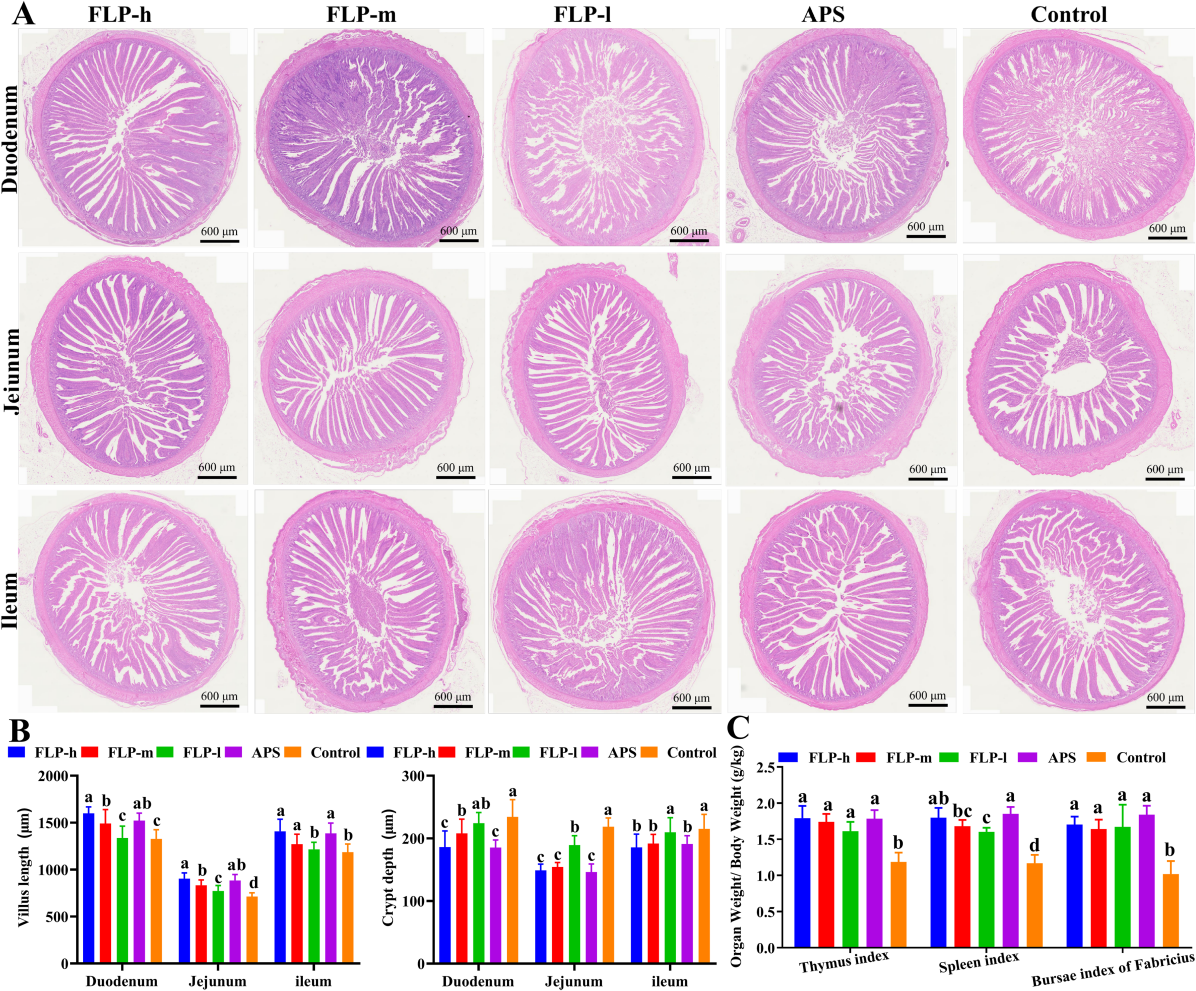
Effect of FLPs on intestinal tissue morphology in chickens. **(A)** The duodenum, jejunum, and ileum were stained with HE (40× scale bar: 600 μm), **(B)** villus height and crypt depth, **(C)** immune organ index. Significant changes are indicated by bars with distinct superscripts **(A–C)** (*p* < 0.05).

As shown in **Figure 3A**; **Supplementary Figure S2**, no discernible pathological changes were observed in the thymus, spleen, bursa of Fabricius, cecal tonsils, duodenum, jejunum, and ileum in all experimental groups. Compared to the control and FLP-l groups (as shown in **Figure 3B**), the crypt depth in the jejunum and ileum was significantly decreased in the FLP-h, FLP-m, and APS groups (*p* < 0.05), while the villus height in the duodenum, jejunum, and ileum was significantly increased in the FLP-h, FLP-m, and APS groups (*p* < 0.05). The results (depicted in **Supplementary Figure S2**) demonstrated that the FLP-h and FLP-m groups had increased thymus cortex area, more compact lymphocytes, increased number of splenic follicles, expanded bursal lymphoid follicles, and more dense and orderly cell arrangement in the endogenous layer of the cecal tonsils, showing excellent immunoactive activity. As depicted in **Figure 3C**, the FLP-h, FLP-m, FLP-l, and APS groups had a significantly increased thymus index, spleen index, and bursa of Fabricius index compared to the control group (*p* < 0.05). No significant differences were observed in the organ index between the FLP-h group and the APS group (*p >*0.05).

### 3.6 Effect of FLPs on goblet cell number and IgA secretion in chicken intestines

**Figure 4:**
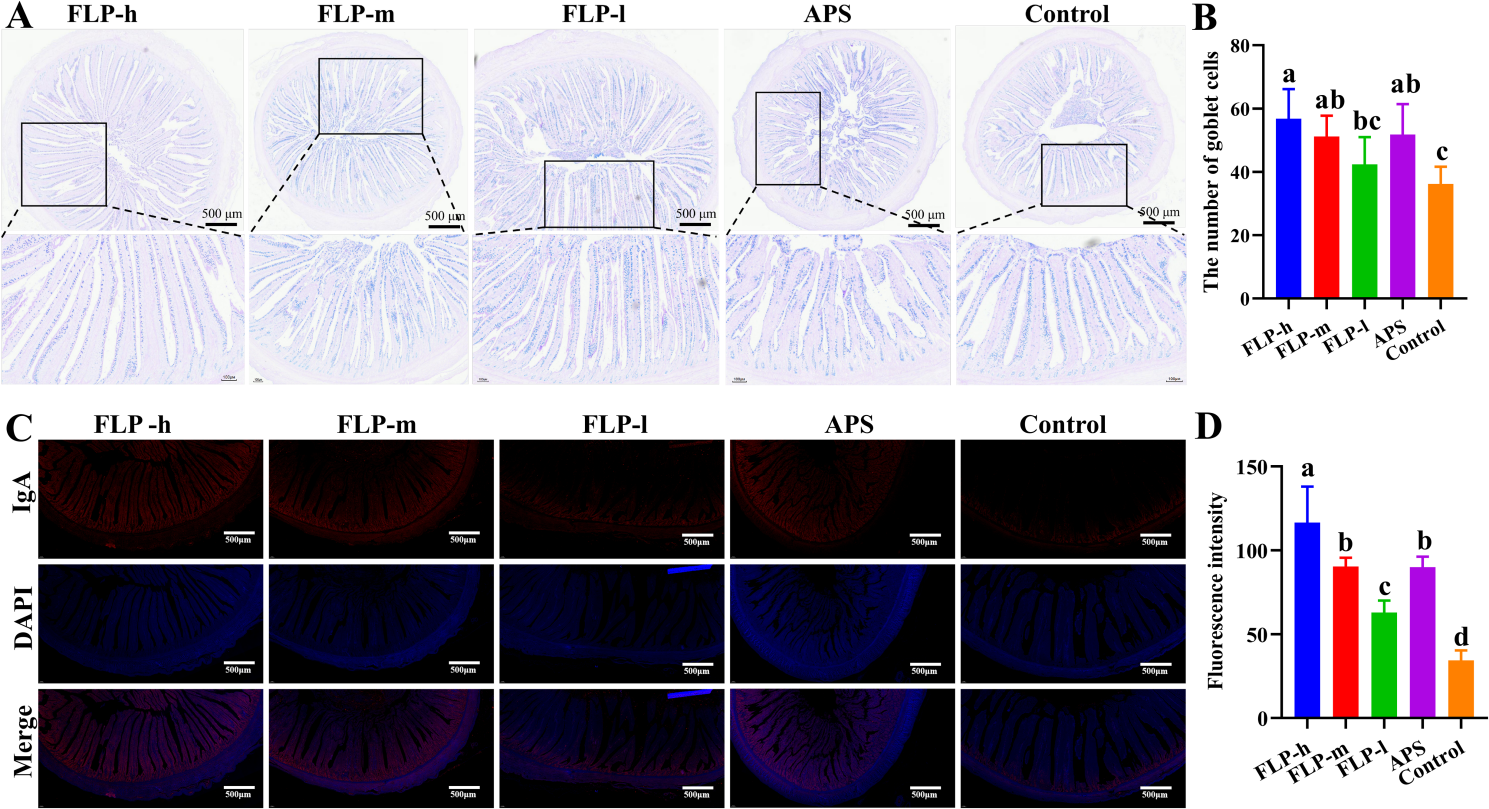
The effects of FLPs on **(A)** goblet cells (40×, 200×, scale bar: 500 μm and 100 μm), **(B)** goblet cell numbers in chicken ileums were observed by AB-PAS staining, **(C)** IgA secretion (100×, scale bar: 500 μm), **(D)** fluorescence intensity was observed by immunofluorescence staining. Significant changes are indicated by bars with distinct superscripts **(A–D)** (*p* < 0.05), n = 5.

The quantity of goblet cells and IgA secretion in the ileum was determined through AB-PAS staining and immunofluorescence to assess the impact of FLPs on the intestinal mucosal barrier function (**Figure 4**). As depicted in **Figures 4A, B**, the FLP-h, FLP-m, and APS groups had a significantly increased number of ileal goblet cells compared to the control group (*p* < 0.05). No significant difference was observed in the number of ileal goblet cells between the control and FLP-l groups (*p* > 0.05).

As depicted in **Figures 4C, D**, the fluorescence intensity of IgA in the FLP-h group was significantly higher than that in the other groups (*p* < 0.05). Both the FLP-m and the APS groups had significantly increased intestinal IgA expression compared to the FLP-m and control groups, and there was no significant difference between the FLP-m and APS groups (*p* > 0.05).

### 3.7 Impact of FLPs on immune function

**Figure 5:**
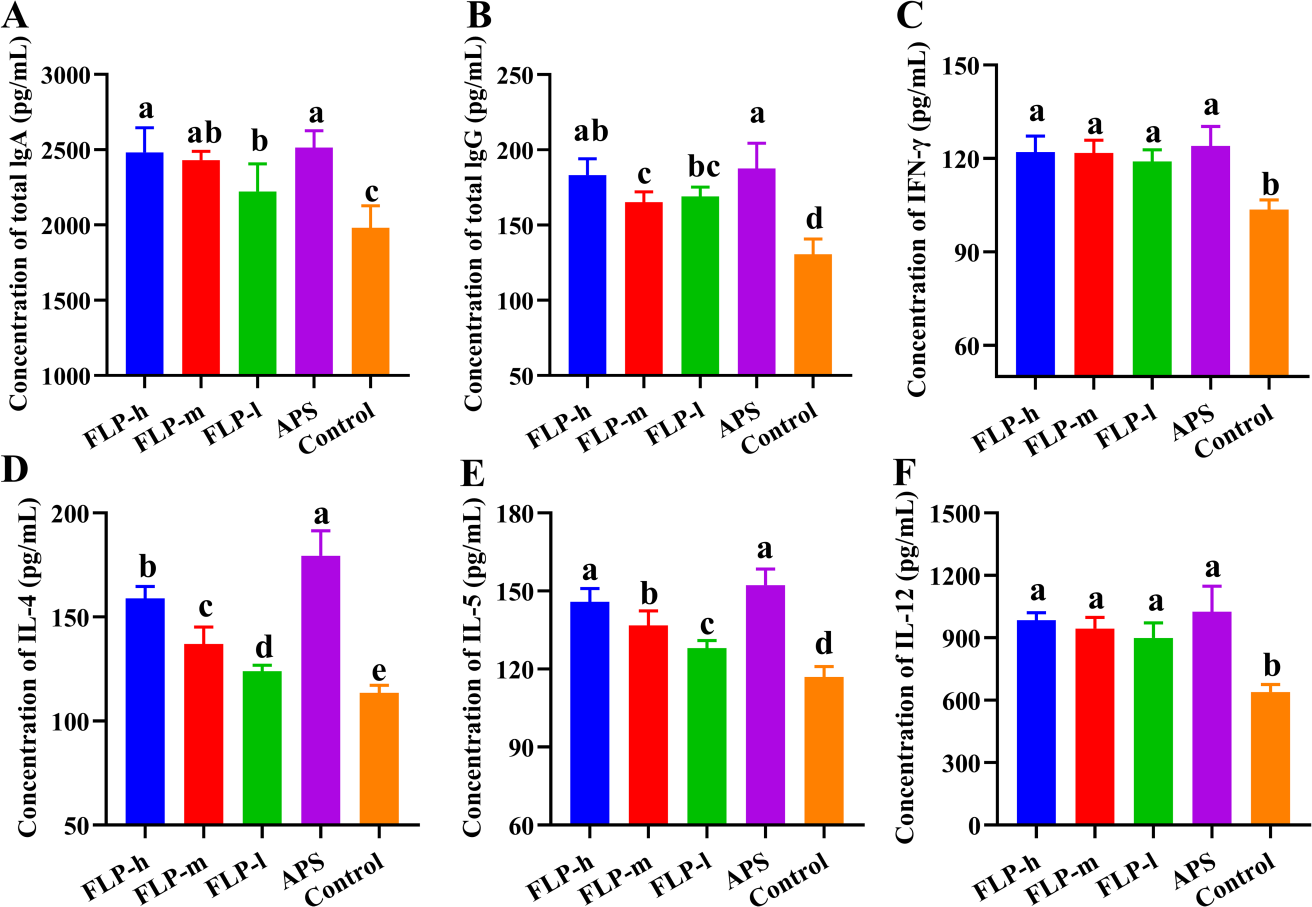
Effect of FLPs on immunoglobulins and cytokines levels. **(A)** IgA, **(B)** IgG, **(C)** IFN-γ, **(D)** IL-4, **(E)** IL-5, and **(F)** IL-12. Significant changes are indicated by bars with distinct superscripts **(A–E)** (*p* < 0.05), n = 5.

Antibodies and cytokines within the immune system collaborate to sustain the body’s immune equilibrium and defense. The regulatory influences of FLP on antibodies and cytokines in the body are presented in **Figures 5A-F**. In comparison with the control group, feeding FLP-h, FLP-m, FLP-l, and APS significantly enhanced the production of sIgA, IgG, IFN-γ, IL-4, IL-5, and IL-12 (*p* < 0.05). Notably, there were no significant disparities in the levels of sIgA, IgG, IFN-γ, IL-5, and IL-12 between the FLP-h group and the APS group (*p* > 0.05).

### 3.8 Effects of FLP on activation of T cell differentiation in spleen

**Figure 6:**
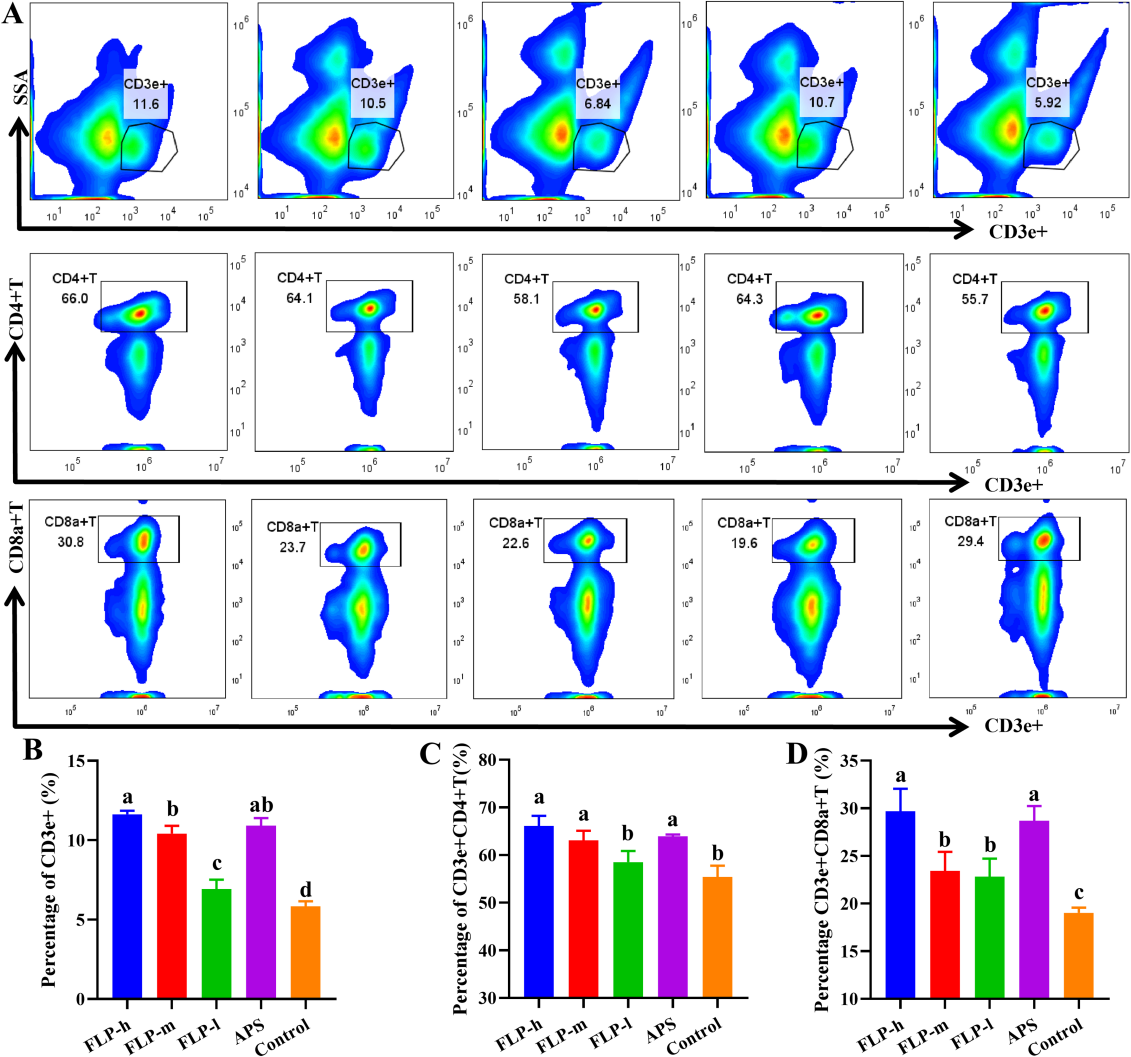
Impact of FLPs on chicken splenic T cell activity. **(A)** CD3e+, CD4+ and CD8a+T-cell differentiation in splenic T-lymphocytes, **(B)** CD3e+ T-cell expression, **(C)** CD4+ T-cell expression, **(D)** CD8a+ T-cell expression. Significant changes are indicated by bars with distinct superscripts **(A–C)** (*p* < 0.05), n = 5.

Determining the presence of CD3e^+^ activation and distinguishing between CD4^+^ and CD8a^+^ T-cells in the spleen following immunization allows us to comprehend the specific immune responses triggered by the immunopotentiator. **Figures 6A–D** illustrate how the activation of spleen CD3e^+^ T, CD4^+^ T, and CD8a^+^ T increased with the concentration of FLPs in the FLP-l, FLP-m, and FLP-h groups. Across every group tested, the FLP-h group and APS group had significantly increased CD3e^+^T, CD4^+^ T, and CD8a^+^ T ratios compared to the FLP-l and control groups (*p* < 0.05).

### 3.9 Effect of FLPs on SCFA

**Figure 7:**
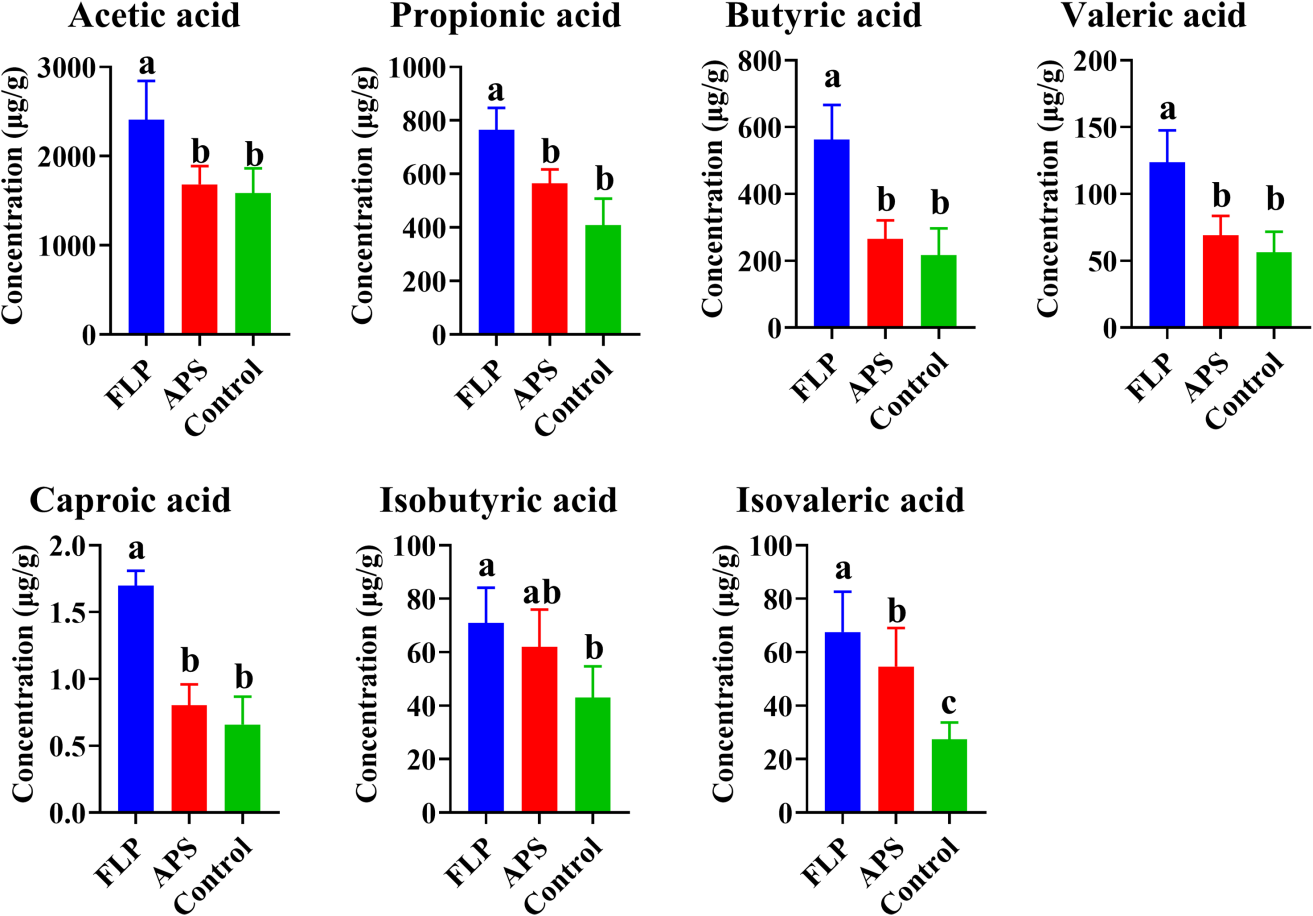
Content of different SCFAs in each group **(A–C)** (*p* < 0.05), n = 5.

SCFAs have a significant impact on preserving the integrity of the intestinal barrier and inhibiting the infiltration of pathogens. Additionally, SCFAs are capable of modulating the immune response through their influence on lymphocyte differentiation. As shown in **Figure 7**, there was a significant increase in acetic acid, propionate, butyrate, valeric acid, propionic acid, and isovaleric acid in the FLP-h group compared to the control and APS groups (*p* < 0.05).

### 3.10 Effects of FLPs on the intestine’s microbes in chickens

**Figure 8:**
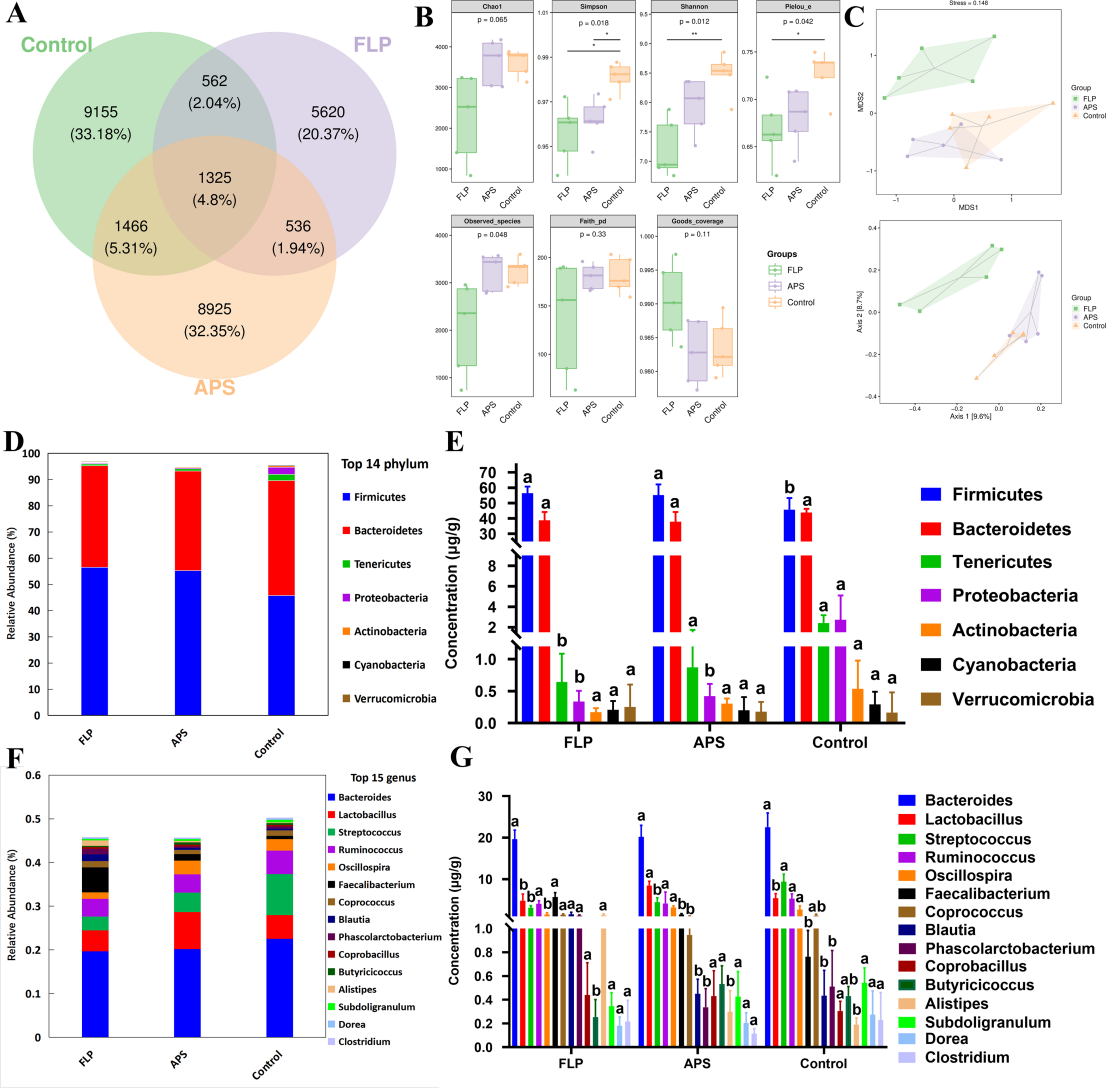
The influence of FLP-h on the composition of microbial communities. **(A)** Venn diagram of the intestine microorganisms. **(B)** Investigation of the intestinal microorganism’s β-diversity. **(C)** Bacterial community principal coordinates analysis (PCoA). **(D)** The phylum level of the main microorganisms in the intestines. **(E)** Intestinal microorganism species composition bar chart at the phylum level. **(F)** Main intestinal microorganisms at the genus level. **(G)** Intestinal microorganism species composition bar chart at the genus level. Bars displaying different superscripts **(A, B)** demonstrate significant variations (*p <*0.05), n = 5.

To assess the impact of the FLP-h treatment on broiler intestinal health in more detail, the abundance and diversity of intestinal microbes in chickens were detected. The gastrointestinal microbial community’s makeup is depicted by the Venn diagram in **Figure 8A**, which shows that the FLP-h group had 5,620 OTUs, the APS group had 8,925, and the untreated control group had 9,155. The FLP-h group and the control group shared 1,887 OTUs, the FLP-h group and APS group shared 1,861 OTUs, and the APS group and control group shared 2,791 OTUs, for a total of 1,325 OTUs. The gut microbiota of chickens was analyzed using α-diversity (**Figure 8B**), which revealed that the FLP-h group had a significantly lower Simpson’s index, Shannon’s index, and Pielou’s evenness index (*p* < 0.05). The intestinal microbial β-diversity analysis findings are displayed in **Figure 8C**. The untreated and APS groups showed full separation from the FLP-h group, with a little overlap between them.

At the phylum levels (**Figures 8D, E**), the FLP-h group exhibited a significantly increased quantity of Firmicutes, and a significantly decreased quantity of Tenericutes and Proteobacteria as compared to the control group (*p* < 0.05). At the genus level (**Figures 8F, G**), the FLP-h group showed a notable rise in the prevalence of *Faecalibacterium, Blautia*, *Phascolarctobacterium*, and *Alistipes* while there was a significant decline in *Streptococcus* and *Oscillospira* compared to the control group (*p* < 0.05).

### 3.11 KEGG pathway prediction analysis

**Figure 9:**
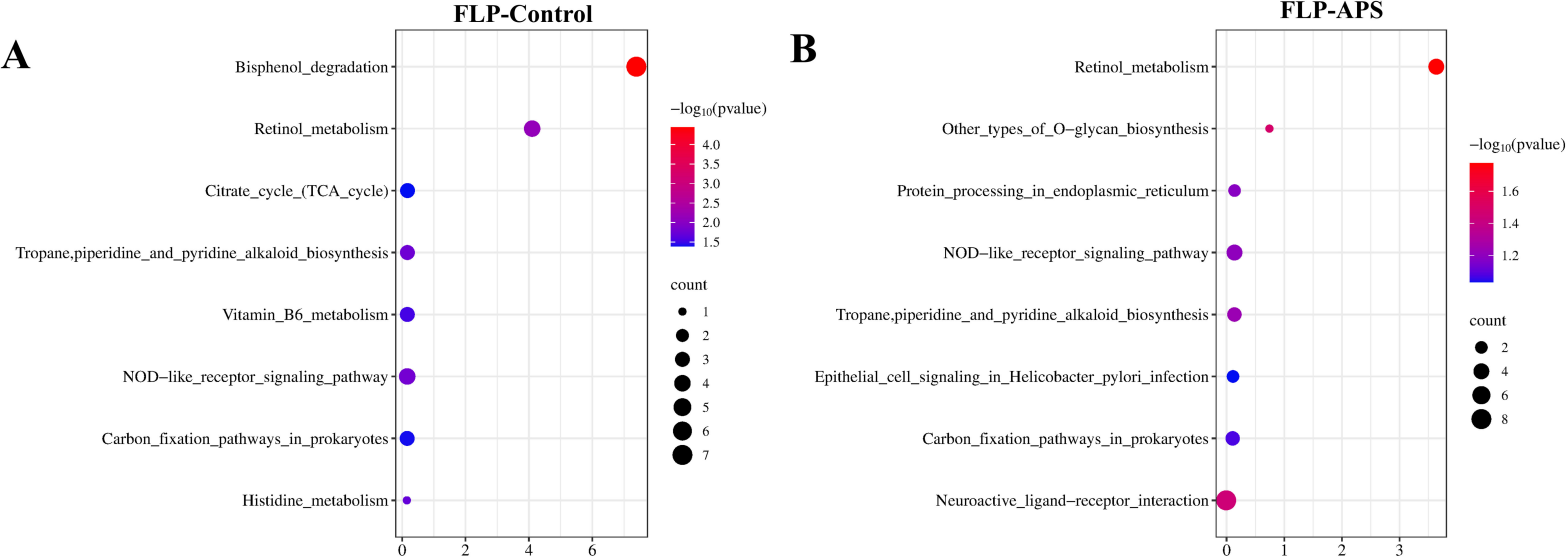
Prediction of the effect of **(A)** FLP group VS control group and **(B)** FLP group VS APS group on KEGG pathways. (n = 5). Note: *p* < 0.05 indicates a significant difference. Section analysis results (40×, 200×, HE, scale bar: 100 μm).

The KEGG pathway was used to predict the immuno-enhancement pathway of FLPs. When comparing with the APS and control groups, it was discovered that eight KEGG pathways were significantly expressed in the FLP group, respectively (**Figures 9A, B**). As shown in **Figure 9A**, the FLP group demonstrated a notable improvement in the breakdown of bisphenol; retinol metabolism; the tropane, piperidine, and pyridine alkaloid biosynthesis pathway; the NOD-like receptor signaling pathway; and carbon fixation pathways in prokaryotes compared to the control group (*p* < 0.05). Similarly, **Figure 9B** indicates that there was a notable improvement from the FLP group in retinol metabolism; NOD-like receptor signaling pathway; the tropane, piperidine, and pyridine alkaloid biosynthesis pathway; and carbon fixation pathway in prokaryotes in comparison with the control group (*p* < 0.05).

## 4 Discussion

Since the use of antibiotics is prohibited in a number of nations, there is a rising interest in creating natural feed additives that can preserve animal health and enhance animal productivity (24). Animal feed additives made from plant-derived polysaccharides are becoming more popular because of their high safety, growth promotion, and immune-modulatory impact (21). Polysaccharides have different biological activities due to their different monosaccharide compositions and structures (25). Therefore, it is important to detect the monosaccharide composition of polysaccharides to predict the activity of polysaccharides. As shown in **Figures 1C–E**, five monosaccharides make up this acid polysaccharide: rhamnose, arabinose, mannose, glucose, and galactose, of which the main components are arabinose and rhamnose. It is known that many acidic polysaccharides have good immune-enhancing activities. Studies have shown that *P. linteus* acidic polysaccharide (PLAP) can improve the repair and protection of colon tissue damage by regulating the level of inflammatory factors, enhancing the secretion of mucosal protective proteins, improving the diversity and species abundance of intestinal microbiota, and increasing the level of beneficial SCFAs (26). Studies have shown that the acid *Lagenaria siceraria* (Molina) Standl polysaccharide (LSP50) was composed of arabinose, rhamnose, and galactose. LSP50 can stimulate the production of high IgG, IFN-g, IL-2, IL-4, and IL-5 in the body; induce the differentiation of spleen CD3e^+^CD8a^+^T cells; and enhance the arachidonic acid pathway to strengthen the immune level of the body (27). In order to preliminarily predict the biological activity of FLPs, the intersection of the monosaccharide action targets of FLPs was conducted using the network pharmacology method, and the co-acting genes and related pathways of these monosaccharides were predicted. As shown in **Figure 2**, FLPs may promote immune enhancement function by controlling the MAPK and toll-like receptor signaling pathways, carbon fixation pathways in prokaryotes, retinol metabolism, and the degradation process of bisphenol A.

In order to detect the biological activity of FLPs, growth performance, slaughtering performance, immune indexes, and intestinal flora of chickens were measured after feeding with FLPs. As depicted in **Tables 1**, **2**, feeding with FLPs leads to a significant increase in the ABW, a decrease in F/G, an increase in slaughter rate, and a reduction in abdominal fat percentage. These findings indicate that FLPs promote growth, improve feed utilization, enhance slaughtering performance, and contribute to the overall meat quality of broiler chickens. A key metric for assessing animal growth rate and attaining financial viability is growth performance (28). It suggests that FLPs have a favorable impact on the growth and development of broiler chickens, improving their performance in terms of growth. The growth performance of animals is directly related to intestinal absorption function and intestinal health. There was a favorable correlation between intestinal absorption and intestinal villi height and defense function, while the depth of intestinal crypts was negatively correlated with absorption and immune function (29). The increased villus height increases the intestinal absorption surface and strengthens the barrier function. The shallower the crypt depth, the better the maturation and secretion ability of crypt stem cells, and the stronger the nutritional absorption and immune enhancement ability (30). As depicted in **Figures 3A, B**, compared with the control and the FLP-l groups, the FLP-h, FLP-m, and APS groups significantly lowered the crypt depth and significantly raised the villus height of the ileum, jejunum, and duodenum (*p* < 0.05). This finding suggested that by improving the villus height and crypt depth structure, FLP-h and FLP-m might enhance the immune system and nutrition absorption performance of the intestine. The mucous layer secreted by goblet cells covering the intestinal epithelium is an important chemical barrier for the intestinal mucosa (31). The results showed that (**Figures 4A, B**) the FLP-h, FLP-m, and APS groups had a significant rise in ileum goblet cell count compared to the control group (*p* < 0.05). Intestinal IgA is an important antibody that regulates intestinal microbes, protects intestinal barrier function, and regulates the mucosal immune system and disease defense (29). The results showed that (**Figures 4C, D**) the FLP-h, FLP-m, and APS groups had a significant rise in intestinal IgA expression compared to the control group (*p* < 0.05). These results suggest that FLP-h and FLP-m can maintain intestinal function by enhancing mucin secretion and intestinal barrier integrity, thereby protecting the intestinal health of chickens.

The intestinal immune barrier is essential for animals to maintain various biological functioning (32). On the gastrointestinal mucosal surface, sIgA functions primarily as a primary defensive antibody, protecting against possible infections (33). The serum and bodily fluids are rich in IgG, which has the ability to attach to antigens present on the surface of pathogens, thereby safeguarding the body from potential harm caused by these pathogens (29). As depicted in **Figure 5**, the FLP group had significantly increased intestinal sIgA and serum IgG production (*p* < 0.05). This implies that FLPs may have the ability to modulate the immune response by enhancing the levels of IgA and IgG, as well as improving the function of the intestinal immune barrier. Cytokines are essential for triggering immunological reactions and eliminating infections in the host’s body (34). The cytokines IL-4, IL-5, IL-10, and IL-12 are involved in the regulation of intestinal mucosa sIgA production, and the cytokines IFN- γ, IL-4, IL-10, and IL-12 are involved in the regulation of serum IgG production (32). According to research, adding polysaccharide feed additives can increase the growth of Th1/Th2 cells and pro-cytokine secretion. In order to alleviate the inflammation brought on by a salmonella infection in broiler chickens, IL-12 stimulates Th1 cell proliferation and IFN-γ synthesis (35, 36). As depicted in **Figure 5**, the FLP group had significantly increased Th 1-type cell secretion factor (IL-12, IFN-γ) and Th 2-type cell secretion factor (IL-4, IL-5). Therefore, it is suggested that FLPs can enhance the body’s immune function and strengthen the intestinal barrier by boosting the production of intestinal sIgA and Th 1-type/Th 2-type cytokines.

The spleen is a vital component of the body’s immune system, containing a large number of T lymphocytes and playing a crucial role in regulating the adaptive immune system (37). CD4^+^ T cells and CD8a^+^ T cells are important surface markers of T cells. CD4^+^ T cells assist the body in producing humoral and cellular immune responses in the immune system and CD8a^+^ T cells have the function of killing target cells. Therefore, the identification of the differentiation and ratio of CD4^+^ T and CD8a^+^ T cells in the spleen can directly indicate the existence and role of helper T cells and cytotoxic T cells in the spleen following stimulation by drug intervention (38). In **Figures 6A–D**, the amount of FLPs in the FLP-m and FLP-h groups enhanced the activation of CD3e^+^ T, CD4^+^ T, and CD8a^+^ T cells in the spleen. When compared to the FLP-l and control groups, the FLP-h group exhibited a substantially higher CD3e^+^ T, CD4^+^ T, and CD8a^+^ T ratio than any other experimental group (*p* < 0.05). These results imply that FLPs have a dose-depending impact on the splenic CD4^+^ T and CD8a^+^ T activation. The FLP-h group outperformed all other experimental groups in terms of splenic T cell activation and humoral immune system regulation.

Among the metabolites produced through bacterial fermentation, SCFAs have been extensively studied and are widely prevalent (39). Research has found that SCFAs can help reduce inflammation in the gut, promote the growth of beneficial bacteria, maintain intestinal health, regulate the immune system, and regulate appetite and body weight (40). As shown in **Figure 7**, there was a significant rise in the acetic acid, propionic acid, butyric acid, valeric acid, caproic acid, and isovaleric acid within the FLP-h group (*p* < 0.05). These findings imply that the inclusion of FLP-h in feed promotes the production of SCFAs in the intestinal tract of broiler chickens. The intestinal tract relies on the intestinal microbiota to protect its physiological and immune functions, promote overall health, and enhance growth performance in broiler chickens (41). This study also aimed to examine variations in microbial communities by incorporating FLP-h into the daily diet. It showed fewer OTUs in the FLP-h and APS groups and significantly lower Simpson’s index, Shannon’s index, and Pielou’s evenness index (**Figures 8A, B**) (*p* < 0.05). These outcomes reveal that the species abundance and diversity of intestine microbes decreased after FLP feeding in broilers, which may be due to the fact that FLPs reduced the number of dangerous bacteria while increasing the number of beneficial species. The β-diversity analysis indicated a clear separation between the FLP-h group from the other two groups (**Figure 8C**). The findings suggest that feeding with FLP-h leads to a decrease in the diversity of intestinal microbiota, and the structure of microbiota is significantly different from the APS and control groups. The decrease in diversity in the FLP-h group may be due to a decrease in harmful bacteria.

This study analyzed the gut microbiota at the phylum and genus levels to further investigate changes in its composition. According to Sun et al. (2023), Firmicutes are capable of breaking down polysaccharides and generating SCFAs. Bacteroidetes can aid in the host’s breakdown of a variety of complex polysaccharides to enhance the absorption of nutrients (42). While Tenericutes and Proteobacteria contain many pathogenic microorganisms (43). The increasing prevalence of Proteobacteria and Tenericutes in the intestine could potentially elevate the risk of pathogenic infections (44). **Figures 8E, G** illustrate that there was a notable rise in the prevalence of Firmicutes in the FLP group, along with a significant reduction in Tenericutes and Proteobacteria compared to the control group (*p* < 0.05). The results suggested that at the phylum level, FLPs have the potential to boost the presence of beneficial bacteria such as Firmicutes, while concurrently reducing harmful bacterial phyla. *Faecalibacterium, Blautia*, and *Phascolarctobacterium* belong to Firmicutes and have various intestinal immune enhancement functions, such as promoting the synthesis of various SCFAs, promoting the growth and repair of intestinal epithelial cells, and exhibiting anti-inflammatory and antioxidant effects (45). *Alistipes* can promote the synthesis of butyric acid and valeric acid, protect the intestinal mucosa, and maintain the function of the intestinal barrier (46). *Streptococcus* and *Oscillospira* also belong to the phylum Firmicutes and an increase in *Streptococcus* may increase the risk of intestinal infection and inflammation (47). The FLP group showed a notable rise in frequency at the genus level of *Faecalibacterium, Blautia, Phascolarctobacterium*, and *Alistipes* and a significant decrease in the abundance of *Streptococcus* and *Oscillospira*. The findings indicate that FLP-h can significantly increase the abundance of Firmicutes at the phylum and genus level, promoting beneficial bacteria in the intestines to become the dominant microbiota, thereby reducing the diversity and abundance of harmful bacteria in the gut.

To further determine the biological activity of FLPs, KEGG pathway analysis was used to predict the immune-enhancing action pathways of FLPs. As depicted in **Figures 9A, B**, the FLP group was significantly enhanced in the bisphenol degradation; retinol metabolism; the NOD-like receptor signaling pathway; the tropane, piperidine, and pyridine alkaloid biosynthesis pathway; and the carbon fixation pathway in prokaryotes compared with control group (*p* < 0.05). Combined with the network pharmacological prediction results of FLPs, the effects of FLPs on the secretion of cytokines and short-chain fatty acids, and the prediction results of the KEGG pathways of FLPs, it can be concluded that FLPs can be effectively utilized by intestinal flora through carbon fixation pathways in prokaryotes. Thus, it can effectively change the structure of intestinal flora and change intestinal metabolites. Moreover, FLP enhances the interaction between the bisphenol degradation pathway, the retinol metabolism pathway, the NOD-like and toll-like receptor signaling pathway, and the MAPK signaling pathway, promoting the intestinal recruitment of B and T cells, regulating dendritic cells and T cells, promoting the secretion of various intestinal cytokines and antibodies, and enhancing the intestinal mucosal barrier function.

## 5 Conclusions

In summary, FLPs are comprised of monosaccharides including rhamnose, arabinose, mannose, glucose, and galactose. Feeding with FLPs can improve the growth performance and slaughtering performance of broilers; increase the production and release of immunoglobulin, cytokines, and short-chain fatty acids; reduce the abundance of pathogenic bacteria; and improve the function of the intestinal mucosal immune barrier through various signaling pathways. Altogether, FLPS can significantly contribute to the improvement of growth performance and intestinal immune function in chickens.

## Data Availability

The datasets presented in this study can be found in online repositories. The names of the repository/repositories and accession number(s) can be found in the article/**Supplementary Material**.
