# Supplementary material for: Effects of Ficus carica L. polysaccharide on the intestinal immune function and microbiota of broilers
Source: Front Immunol. 2025 Apr 8;16:1579046. doi: 10.3389/fimmu.2025.1579046 (PMC12011799; doi:10.3389/fimmu.2025.1579046)
Supplement: Supplementary file 1 [file Table1.docx]

**Table Chromatographic condition**

| Time | A（%） | B（%） |
| --- | --- | --- |
| 0 | 5 | 95 |
| 15 | 20 | 80 |
| 25 | 30 | 75 |
| 35 | 100 | 0 |
| 40 | 100 | 0 |
| 45 | 5 | 95 |
| 50 | 5 | 95 |

Chromatographic column: Agilent Eclipse XDB C18 (4.6 x 250 mm, 5 µm)

Elution condition

A: Acetonitrile; B: Water

Processing software: TCM Chromatographic Fingerprint Similarity Evaluation System (2012.130723 edition)

**Table The result of similarity calculation**

|  | *Ficus carica L*. (S1) | Standard goods (R3) |
| --- | --- | --- |
| *Ficus carica L*. (S1) | 1.000 | 1.000 |
| Standard goods (R3) | 1.000 | 1.000 |


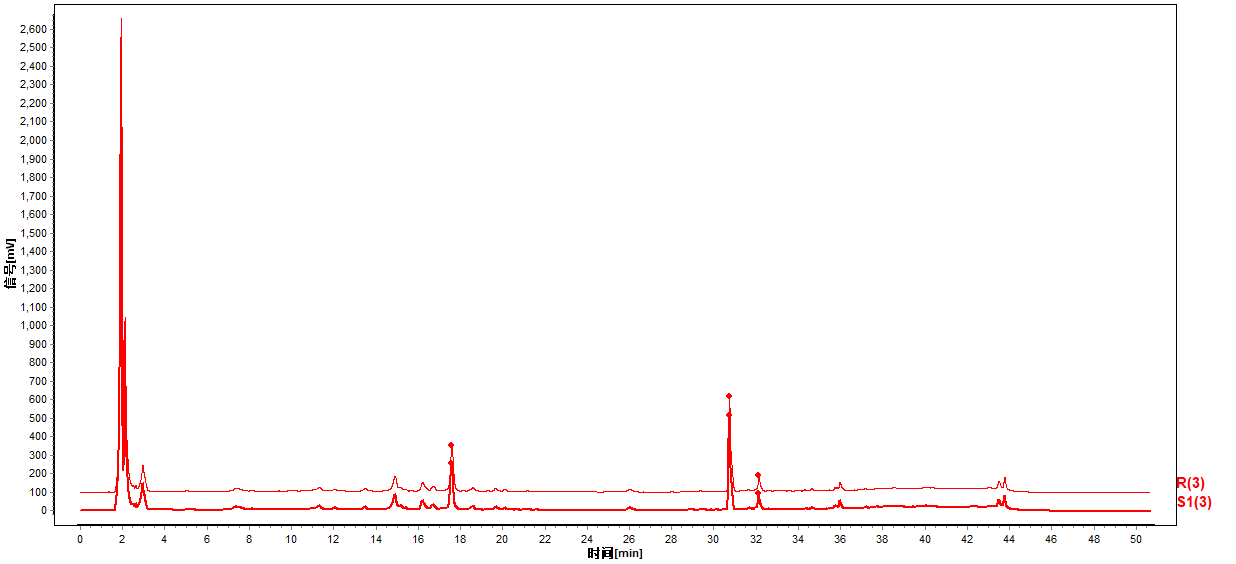


**Fig. S 1 Fingerprint**

*Ficus carica L*. samples were evaluated by TCM chromatographic fingerprint similarity evaluation system (2012.130723 edition) with a time width of 0.1. Automatic matching was adopted to generate control map R, and a total of 11 peaks were determined. Through the similarity calculation, it can be concluded that the similarity is 100%.


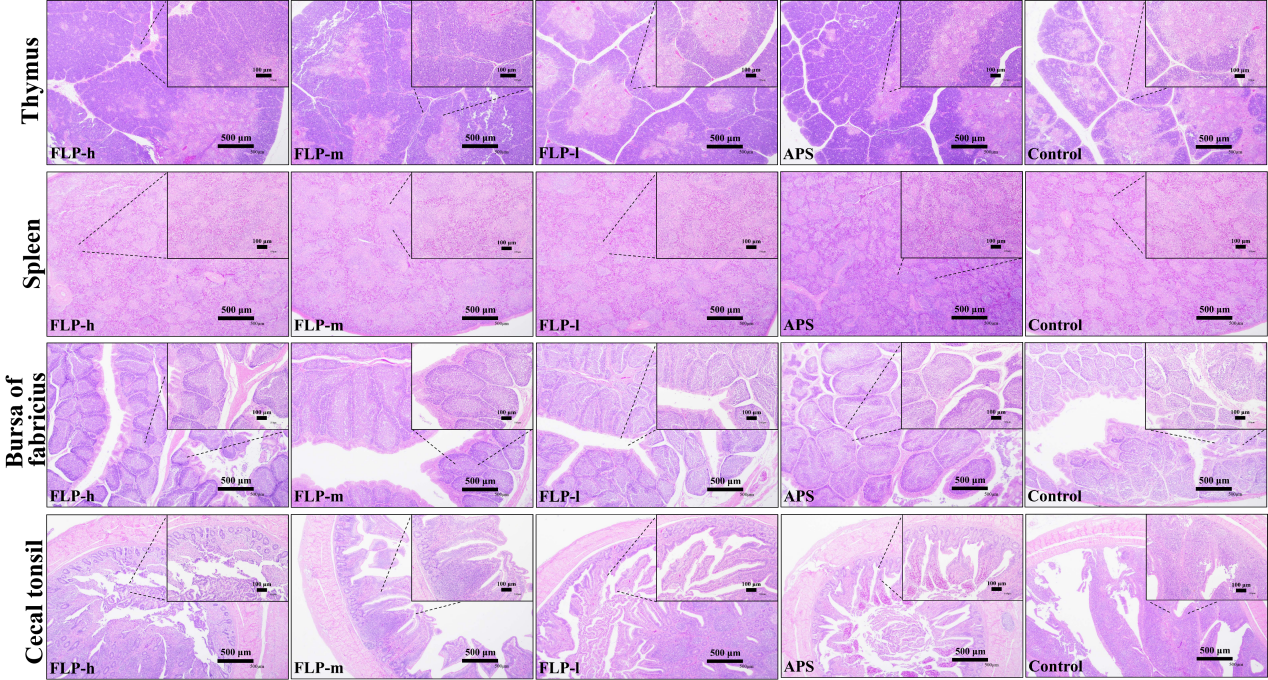


**Fig. S 2 Tissue Section Analysis Results (40×, 200×, HE, scale bar: 100 μm).**
